# Novel Doxorubicin Derivatives: Synthesis and Cytotoxicity Study in 2D and 3D *in Vitro* Models

**DOI:** 10.15171/apb.2017.071

**Published:** 2017-12-31

**Authors:** Roman Akasov, Maria Drozdova, Daria Zaytseva-Zotova, Maria Leko, Pavel Chelushkin, Annie Marc, Isabelle Chevalot, Sergey Burov, Natalia Klyachko, Thierry Vandamme, Elena Markvicheva

**Affiliations:** ^1^Polymers for Biology Laboratory, Shemyakin-Ovchinnikov Institute of Bioorganic Chemistry of the Russian Academy of Sciences, 117997, Miklukho-Maklaya 16/10, Moscow, Russia.; ^2^Institute of Molecular Medicine, Sechenov First Moscow State Medical University, 119991, Trubetskaya str. 8-2, Moscow, Russia.; ^3^Institute for Regenerative Medicine, Sechenov First Moscow State Medical University, 119991, Trubetskaya str. 8-2, Moscow, Russia.; ^4^Synthesis of Peptides and Polymer Microspheres Laboratory, Institute of Macromolecular Compounds of the Russian Academy of Sciences, 199004, Bolshoi pr. 31, Saint-Petersburg, Russia.; ^5^UMR CNRS 7274, Laboratoire Réactions et Génie des Procédés, Université de Lorraine, 54518, 2 avenue de la Fort de Haye, Vandoeuvre lès Nancy, France.; ^6^Faculty of Chemistry, Lomonosov Moscow State University, 119991, Leninskiye Gory 1-3, Moscow, Russia.; ^7^CNRS UMR 7199, Laboratoire de Conception et Application de Molécules Bioactives, University of Strasbourg, 74 route du Rhin, 67401 Illkirch Cedex, France.

**Keywords:** Aantitumor drug screening assays, Microencapsulation, Multicellular spheroids, Multiple drug resistance, Serum albumin

## Abstract

***Purpose:*** Multidrug resistance (MDR) of tumors to chemotherapeutics often leads to failure of cancer treatment. The aim of the study was to prepare novel MDR-overcoming chemotherapeutics based on doxorubicin (DOX) derivatives and to evaluate their efficacy in 2D and 3D in vitro models.

***Methods:*** To overcome MDR, we synthesized five DOX derivatives, and then obtained non-covalent complexes with human serum albumin (HSA). Drug efficacy was evaluated for two tumor cell lines, namely human breast adenocarcinoma MCF-7 cells and DOX resistant MCF-7/ADR cells. Additionally, MCF-7 cells were entrapped in alginate-oligochitosan microcapsules, and generated tumor spheroids were used as a 3D in vitro model to study cytotoxicity of the DOX derivatives.

***Results:*** Due to 3D structure, the tumor spheroids were more resistant to chemotherapy compared to monolayer culture. DOX covalently attached to palmitic acid through hydrazone linkage (DOX-N_2_H-Palm conjugate) was found to be the most promising derivative. Its accumulation levels within MCF-7/ADR cells was 4- and 10-fold higher than those of native DOX when the conjugate was added to cultivation medium without serum and to medium supplemented with 10% fetal bovine serum, respectively. Non-covalent complex of the conjugate with HSA was found to reduce the IC_50_ value from 32.9 µM (for free DOX-N_2_H-Palm) to 16.8 µM (for HSA-DOX-N_2_H-Palm) after 72 h incubation with MCF-7/ADR cells.

***Conclusion:*** Palm-N_2_H-DOX conjugate was found to be the most promising DOX derivative in this research. The formation of non-covalent complex of Palm-N_2_H-DOX conjugate with HSA allowed improving its anti-proliferative activity against both MCF-7 and MCF-7/ADR cells.

## Introduction

Doxorubicin (DOX) is an anthracycline antibiotic which is widely used to treat hematological malignancies, carcinomas, and soft tissue sarcomas since early 1980^th^. The major molecular mechanisms responsible for direct anticancer DOX effects include inhibition of topoisomerase II, nuclear DNA damage, and induction of reactive oxygen species.^1-3^ However, there are some limitations of DOX cancer therapy, including lack of solubility, rather poor biodistribution, and non-specific action leading to cardiac and renal toxicity.^4^ Additionally, in response to anticancer DOX therapy, multidrug resistance could be developed.^5,6^ The resistance of tumor cells to DOX could be mediated through various pathways, including physiological factors (e.g. interstitial fluid pressure in tumors, diffusion limitations, hypoxia, etc.) and cellular factors which are generally associated with overexpression of ATP-binding cassette efflux transporters in cancer cells.^7,8^ Moreover, the physiological characteristics of tumor tissue, such as hypoxia, low nutrient supply, and low pH have been suggested to upregulate the expression of MDR proteins through specific cellular signaling pathways.^9^

To overcome a resistance of cancer cells against DOX-based drugs, chemical modification of the DOX molecule is a commonly used strategy. To date, a number of approaches have been proposed, including prodrug strategy^10^ and DOX encapsulation in nanosized vehicles, such as liposomes, emulsions, polymeric micelles, etc.^11^ Recently, the conjugates of HSA covalently attached to DOX have been proposed as a drug carrier which allowed to improve pharmacokinetic profile and to increase drug accumulation in tumors due to the enhanced permeability and retention (EPR) effect.^12,13^ To this end, the DOX molecule was either covalently attached to the exogenous albumin using a pH-dependent bond,^14^ or there was a linker cleavable with enzymes in tumor tissue.^15^ An alternative approach is based on the DOX derivatives conjugation with endogenous albumin directly in the bloodstream. This approach has been used to obtain aldoxorubicin which is (6-maleimidocaproyl) hydrazone of DOX.^16^ In the current study, we combined the approaches mentioned above. First, we obtained a set of DOX derivatives (with palmitic acid, 5-fluorouracil, 4-carboxybutyl-triphenylphosphonium bromide and aminoguanidine), then we used the most promising drug candidate for non-covalent complex formation with exogenous HSA, in order to improve drug solubility in aqueous media and to provide EPR-based targeting.

Since physiological characteristics as well as cell-cell and cell-matrix interactions could not be properly represented in conventional two-dimensional (2D) cell monolayer culture, a number of three-dimensional (3D) systems have been proposed.^17^ Currently, the most widely used 3D *in vitro* model is based on multicellular tumor spheroids (MTS), which were proposed in the early 70^th^ by Sutherland^18^ and then were used as an excellent tool to recapitulate *in vivo*-like growth of solid tumors.^19^ To generate tumor spheroids, we used semi-permeable alginate-oligochitosan microcapsules which allowed us to obtain MTS with a desired mean size (200-600 µm) and narrow size distribution as described earlier.^20^

The aim of the study was to prepare novel MDR-overcoming chemotherapeutics based on DOX derivatives and to evaluate their efficacy in 2D and 3D *in vitro* models, namely monolayer cell culture and microencapsulated tumor spheroids.

## Materials and Methods

### Reagents

Sodium alginate (medium viscosity, 3500 cps at 25°C), Calcium chloride (CaCl_2_×2H_2_O), EDTA sodium salt, MTT (Thiazolyl Blue Tetrazolium Bromide, 98%), Hoechst 33342, Calcein AM, Propidium iodide (PI), ﬂuorophor protector CC/Mount, human serum albumin were purchased from Sigma-Aldrich. MitoTracker Orange was from Thermo Fisher Scientific. Dimethyl sulfoxide (DMSO), phosphate buffered saline (PBS, pH 7.4), Dulbecco’s modiﬁed Eagle’s medium (DMEM), L-glutamine, sodium pyruvate, Penicillin-Streptomycin, and 2-mercaptoethanol were from PanEko (Russia) and fetal bovine serum (FBS) was from PAA (Austria). All reagents for DOX derivatives synthesis were purchased from JSC ONOPB, CJSC Veropharm, Iris Biotech GMBH and Sigma-Aldrich. Solvents were purified according to the standard protocols. Oligochitosan (Mw 3400 Da, DD 87%) was prepared as described previously^21^ and kindly provided by Prof. A. Bartkowiak (Poland).

### Synthesis of DOX derivatives

#### Palmitoyl-hydrazone of doxorubicin (Palm-N_2_H-DOX)

A solution of Palm-N_2_H_3_ (77 mg, 0.2 mmol) and trifluoroacetic acid (TFA) (30 µl, 0.4 mmol in 5 mL of methanol) were added to a DOX*HCl solution (12 mg, 0.02 mmol) and TFA (10 µl 0.13 mmol in 10 mL of methanol) at stirring. The obtained reaction mixture was stirred for 8 h in the darkness. Then the solvent was partially evaporated under a reduced pressure, while the obtained product was precipitated with acetonitrile, filtered and washed with methyl tert-butyl ether (MTBE). The yield of Palm-N_2_H-DOX was 15 mg (94%).

#### N-palmitoyl-doxorubicin (N-Palm-DOX)

N-hydroxysuccinimide ester of palmitic acid (66 mg, 0.187 mmol) and N,N-diisopropylethylamine (65 µl, 0.374 mmol) were added to the DOX*HCl solution (100 mg, 0.17 mmol) in 2 mL of N,N-dimethylformamide (DMF) at stirring. The reaction mixture was stirred for 18 h in the darkness. The solvent was evaporated under the reduced pressure, while the obtained product was precipitated with water and filtered. The obtained precipitate was purified by chromatography on silica gel (Sigma, 60A, 230-400 mesh) using a chloroform : methanol mixture (10 : 1, v/v) as an eluent. Then fractions containing the final product were combined and evaporated. The yield of N-Palm-DOX was 56 mg (42%).

#### DOX conjugate with the hydrazide of 1-carboxy-5-fluorouracil (DOX-5FU)

DOX*HCl (38 mg, 0.065 mmol) and TFA (3 µl, 0.04 mmol) were added to 1-carboxymethyl-5-fluorouracil hydrazide (40 mg, 0.13 mmol) in 12 ml of absolute methanol at stirring. The reaction mixture was stirred in the darkness for 24 h at room temperature, then it was concentrated in vacuo to 3 ml, and 9 ml of acetonitrile was added. After cooling for 12 h at 0°C, the obtained precipitate was filtered and purified by phase reverse HPLC using System Gold instrument (Beckman, USA) and YMC-Triart C18 column (5 µm, 250 x 10 mm). The mobile phase consisted of acetonitrile/water (85:15 v/v). Flow rate was fixed at 1 ml/min for analytical and 10 ml/min for preparative chromatography. Detection was performed by UV spectroscopy at 220 nm. The fractions containing the final product were combined and evaporated under the reduced pressure. The yield of DOX-5FU was 14 mg (15%).

#### DOX conjugate with (4-carboxybutyl)triphenylphosphonium bromide (DOX-TPP)

1-ethyl-3-(3-dimethylaminopropyl)carbodiimide (43 mg, 0.22 mM) was added to solution of (C_6_H_5_)_3_P(Br)(CH_2_)_4_COOH (100 mg, 0.22 mM) and p-nitrophenol (32 mg, 0.22 mM) in DMF at stirring (0^º^C). The reaction mixture was stirred for 1 h, then the DOX*HCl solution (58 mg, 0.1 mmol) and triethylamine (30 µl, 0.2 mmol) were added. The mixture was stirred for 24 h at room temperature in the darkness, then the solvent was partially evaporated under the reduced pressure, and the obtained product was precipitated with MTBE. The precipitate was filtered and purified using column chromatography on Chemapol silica gel 100/160 µm. The yield of the DOX-TPP was 38 mg (42%).

#### DOX conjugate with aminoguanidine (DOX-AMG)

To the stirred DOX*HCl solution (27.3 mg, 47 µmol) in methanol (9 ml) with TFA catalytic amount (36 µl) aminoguanidine bicarbonate (36 mg; 265 µmol) was added, and the obtained reaction mixture was stirred for 6 days at room temperature in the darkness. When upon 90% of conversion was reached (as confirmed by analytical RP-HPLC), the reaction mixture was evaporated. The residue was dissolved in acetate buffer (pH 5.2) and purified using preparative RP-HPLC. The fractions containing the final product were combined and freeze-dried. The yield of DOX-AMG was 25 mg (88%).

#### Preparation of HSA complexes with Palm-N_2_H-DOX and N-Palm-DOX

A solution of DOX derivative (0.4 mg, 0.48 mmol) in 0.4 ml of DMSO was added to a HSA solution (30.3 mg, 0.45 mmol) in 2 ml of H_2_O and 1 ml of DMSO. The final product was purified by dialysis against water using a 12–14 kDa cut-off dialysis tubing (Orange Scientific, Belgium). The obtained solution was filtered and lyophilized.

#### Cells

Two human breast adenocarcinoma cell lines, namely wild-type MCF-7 and DOX-resistant MCF-7/ADR cells, were kindly provided by Prof. V. Akatov (Institute of Theoretical and Experimental Biophysics Rus Acad Sci, Pushchino, Moscow region, Russia). The cells were cultured in DMEM supplemented with 10% FBS, 2 mM glutamine, 1 mM sodium pyruvate, 100 µg/mL streptomycin, 100 U/mL penicillin, and 50 μM 2-mercaptoethanol in CO_2_-incubator (HERAEUS B5060 EK/CO_2_) at 37°C.

#### Cell microencapsulation and cultivation

Cell microencapsulation was carried out as described earlier.^20^ Briefly, the cells were added to a sterile sodium alginate solution (1.5% w/v, 10^6^ cells per ml), and the mixture was dropped into a CaCl_2_ solution (0.5% w/v) using an electrostatic bead generator. To form an alginate-oligochitosan membrane, the obtained Ca-alginate microbeads were incubated in an oligochitosan solution (0.2% w/v) for 10 min. Then the beads were washed with a 0.9% NaCl solution, and treated with a 50 mM EDTA solution for 10 min, in order to dissolve a Ca-alginate core. Finally, the obtained microcapsules were washed 3 times with 0.9% NaCl solution, transferred to culture flasks with DMEM (10% FBS) and placed into a CO_2_-incubator. All solutions for cell microencapsulation were prepared in the 0.9% NaCl solution. The microencapsulated cells were cultivated for 1-3 weeks. Cell growth in the microcapsules was observed using light microscopy (Reichert Microstar 1820E, Germany).

#### Live-dead assay of the microencapsulated cells 

The microencapsulated tumor spheroids were stained with Calcein AM (50 µM, 30 min) and PI dyes (50 µM, 10 min), in order to visualize alive and dead cells, respectively. The stained spheroids were studied using Leica TCS SP confocal scanning system (Leica, Germany), excitation/emission wavelength were 488 nm/500-530 nm for Calcein AM and 543 nm/560-650 nm for PI.

#### Cytotoxicity study

All DOX derivatives and conventional DOX were dissolved in DMSO to get final concentration of 10 mM, except HSA complexes that were soluble in serum free DMEM. The stock solutions were stored at -20°C. All appropriate working dilutions in cell culture medium were prepared immediately prior to testing.

Cytotoxicity of the DOX derivatives was studied using both monolayer culture and microencapsulated spheroids. To form cell monolayer, the cells were seeded into 96-well plate (5000 cells/well) and incubated overnight; after that monolayer was exposed to 0.001-0.2 mM of the DOX derivatives in 100 µl of DMEM (10% FBS) per well for 24, 48 and 72 h. Aliquots of the microencapsulated spheroids (25 µl of slurry) were added into each well of 96-well plates and incubated with DOX derivatives for 48 and 72 h. Cell viability was assessed using MTT assay. Briefly, the cells were incubated in 100 µL DMEM containing 0.5 mg/ml MTT for 4 h, and then the medium was replaced with 100 µl of DMSO, in order to dissolve the formed formazan crystals. The absorbance (540 nm) was read with an absorbance plate reader (Thermo Scientific, Multiskan FC, USA). Cell viability after the treatment was calculated according to the equation: (OD sample – OD background)/(OD control – OD background) × 100%. The cells without treatment were used as controls. A half maximal inhibitory concentration (IC_50_) was determined as a drug concentration which resulted in 50% inhibition of cell growth.

#### Assessment of the intracellular DOX distribution

The cells were seeded on a cell culture 8-well glass slide (50000 cells per well) followed by an overnight incubation. Then the cells were incubated with the DOX derivative solutions (100 µM, 250 µl per each well) in serum free medium in the CO_2_-incubator for 30 min. The cells were additionally stained with Hoechst 33342 (50 µM, 10 min) and Calcein AM (25 µM, 15 min) for nuclei and cytoplasm visualization, respectively. In some experiments, in order to visualize mitochondria, the cells were stained with MitoTracker Orange (500 nM, 30 min). Finally, the cells were washed three times with PBS, mounted in the CC/Mount ﬂuorophor protector, and observed using Leica CTR 6500 confocal microscope (Germany). The excitation wavelengths were 360 nm for Hoechst, 488 nm for Calcein AM, and 543 nm for DOX derivatives or MitoTracker Orange. Fluorescence signals were collected at 380-460 nm for Hoechst, 500-530 nm for Calcein AM, and 560-650 nm for DOX or MitoTracker Orange. The images were processed in Image J software.

#### Assessment of DOX derivative cellular uptake

To carry out flow cytometry analysis, the BD FACSCalibur fluorescent-activated flow cytometer and the BD CellQuest software were used. The cells were seeded in 24-well cell culture plates (50000 cells per well) and incubated overnight. Then the media was removed, and DOX derivatives were added to the cells (100 µM, 250 µl per each well), in serum free DMEM or DMEM supplemented with 10% FBS. After 30 min of incubation, the cells were washed three times with PBS (pH 7.4), detached with 0.02% EDTA-trypsin solution, and analyzed by flow cytometry with at least 10 000 cells being measured in each sample. The uptake level was determined as a median fluorescence intensity of each sample in relation to a median fluorescence intensity of the control (non-treated cells).

## Results

**Figure 1:**
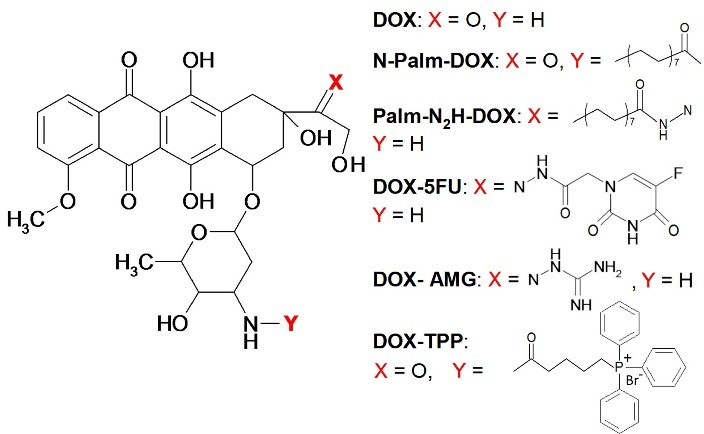
Structure of the doxorubicin (DOX) and its derivatives modified with palmitic acid (N-Palm-DOX and Palm-N_2_H-DOX), 5-fluorouracil (DOX-5FU), aminoguanidine (DOX-AMG), and triphenylphosphonium bromide (DOX-TPP).

In this study, five DOX derivatives have been synthesized (Figure 1). Molecular weights were confirmed by ESI mass spectrometry. An ability of the DOX derivatives to overcome MDR was characterized *in vitro* using MCF-7/ADR human breast cancer cell subline (resistant to DOX) and the parent MCF-7 cell line (susceptible to DOX).

### Intracellular localization of the DOX derivatives

**Figure 2:**
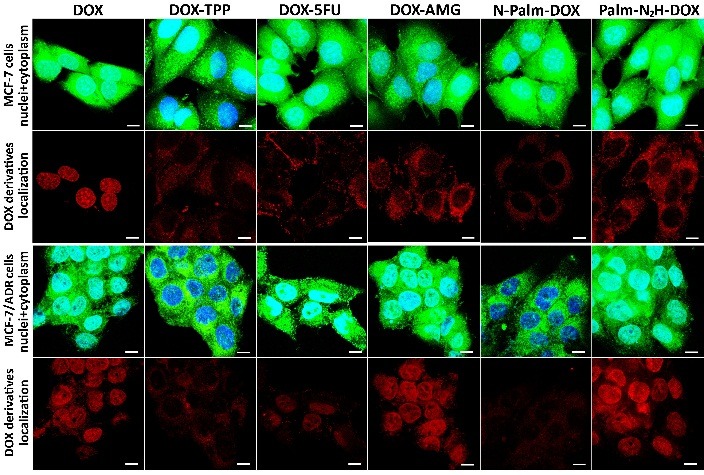
Confocal images of MCF-7 and MCF-7/ADR cells after treatment with various DOX derivatives for 30 min at 37°C. Cell nuclei were stained with Hoechst 33342 (in blue) and cytoplasm was stained with Calcein AM (in green). Scale bar is 10 µm.

Intracellular localization of the DOX derivatives was assessed by confocal microscopy (Figure 2). It was found that DOX modification did affected drug localization in the cells. For instance, in MCF-7 cells free DOX was found to accumulate in the cell nuclei, while all the obtained derivatives were observed outside the nuclei. The localization of native DOX and DOX derivatives in the MCF-7/ADR and MCF-7 cells differed. Thus, in MCF-7/ADR cells DOX was mostly accumulated in the nuclei and partially in the cytoplasm. Similar tendency was revealed for DOX-5FU, DOX-AMG and Palm-N_2_H-DOX derivatives, while DOX-TPP and N-Palm-DOX were localized outside the nucleus.

### Study of accumulation of the DOX derivatives within the cells

**Figure 3:**
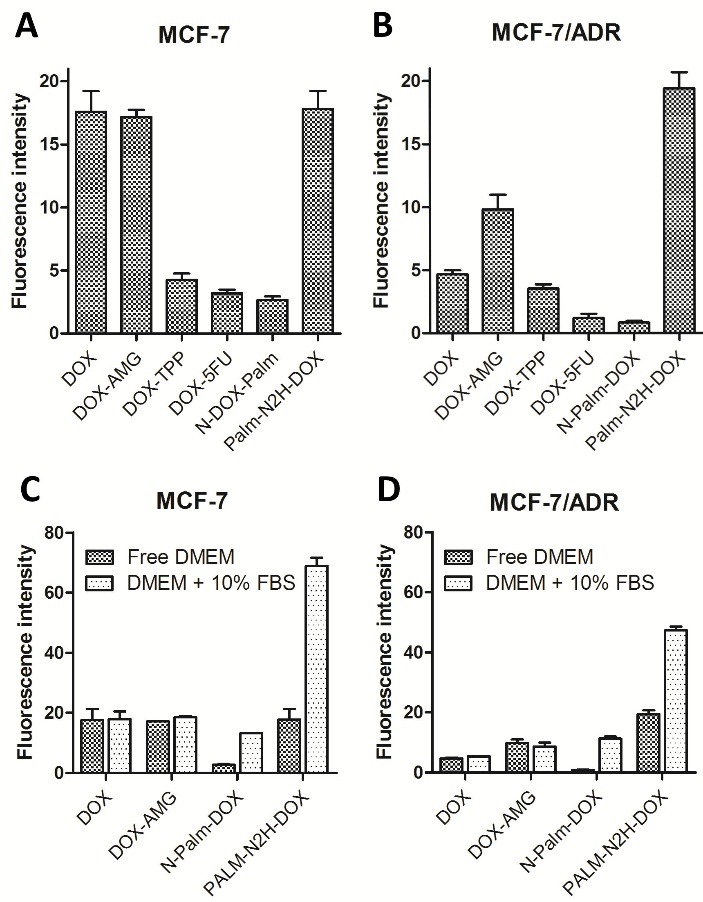
Accumulation of the DOX derivatives in the MCF-7 (A, C) and MCF-7/ADR (B, D) cells. The cells were incubated with DOX derivatives in serum free medium (A, B) and in DMEM supplemented with 10% FBS (C, D) for 30 min at 37°C. Data are expressed as the median fluorescent intensity divided to the background intensity of the control (non-treated cells).

The intracellular accumulation of the DOX derivatives was measured by flow cytometry (Figure 3). As it was expected, DOX uptake by resistant MCF-7/ADR cells was 3.8-fold lower than that by the wild type MCF-7 cells. The accumulation values of DOX-AMG and Palm-N_2_H-DOX conjugates were similar to that of native DOX in MCF-7 cells, while the uptake levels of these derivatives in MCF-7/ADR cells were 2.1 and 4.1-fold higher, respectively. The uptake levels of other three DOX derivatives were significantly lower for both MCF-7 and MCF-7/ADR cell lines. It should be noted that these results were obtained in serum free medium. An addition of 10% FBS to culture medium led to well pronounced changes in the DOX derivatives uptake levels only in the case of two palmitic acid-based conjugates, namely N-Palm-DOX and Palm-N_2_H-DOX. In the complete DMEM (10% FBS) these conjugates were found to accumulate in both MCF-7 and MCF-7/ADR cells more intensively than in serum free medium (Figure 3 C, D). For instance, the cellular uptakes of the Palm-N_2_H-DOX and N-Palm-DOX conjugates by MCF-7/ADR cells in complete DMEM were 2.5-fold and 13.3-fold higher, respectively, than those in serum free medium.

### Cytotoxicity study using monolayer culture

Cytotoxicity study of all DOX derivatives was evaluated by MTT assay in monolayer culture for both MCF-7 and MCF-7/ADR cell lines (Table 1). In all cases the MCF-7/ADR cells were found to be more resistant to drug treatment than MCF-7 cells. As seen in Table 1, although the use of native DOX allowed to get IC_50_ values which were lower than those of the DOX derivatives, all of these derivatives could be considered as MDR overcoming drug candidates in terms of a resistance index (RI). The RI was calculated according to the following equation: R = (IC_50_ of MCF-7/ADR cells) / (IC_50_ of MCF-7 cells).

**Table 1:** The IC_50_ values of native DOX and the DOX derivatives in monolayer culture.

**Samples**	**IC**_50_ **, µM**						**RI** **(72h)**
	**MCF-7 cells**			**MCF-7/ADR cells**			
	**24 h**	**48 h**	**72 h**	**24 h**	**48 h**	**72 h**	
**DOX**	1.50	0.51	0.35	55.1	19.7	14.3	40.8
**Palm-N**_2_ **H-DOX**	24.9	8.3	2.9	85.6	46.0	32.9	11.3
**N-Palm-DOX**	84.1	10.3	4.9	>200	170.8	157.5	32.1
**DOX-5FU**	10.5	8.5	7.1	214.5	187.5	172.7	24.3
**DOX-AMG**	133.1	38.2	22.1	162.2	104.3	76.5	3.5
**DOX-TPP**	>200	41.8	20.2	>200	115.6	83.3	4.1

### Generation of the tumor spheroids in the microcapsules

**Figure 4:**
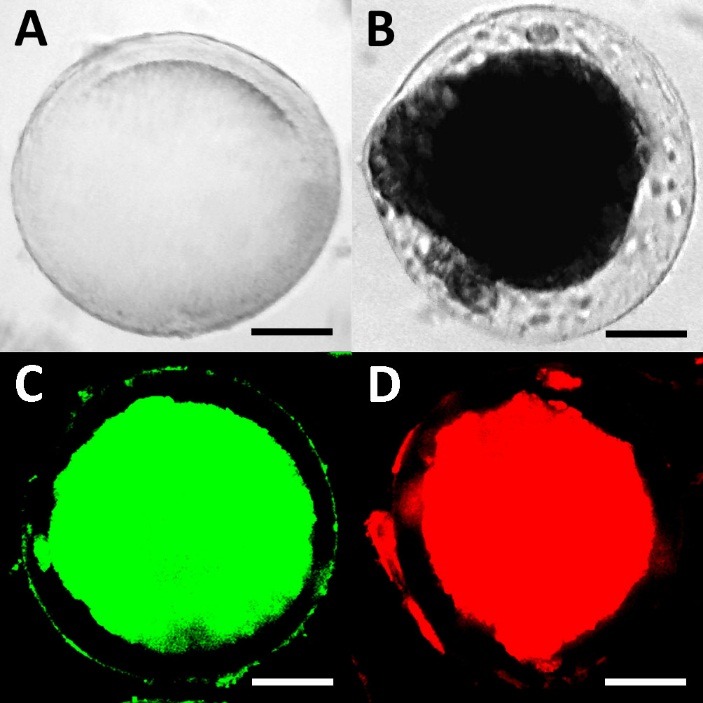
The microphotographs of alginate-oligochitosan microcapsules used for generation of tumor spheroids: the microcapsule without cells (A); the microcapsule with the spheroid from MCF-7 cells after 14 days of cultivation (B); the spheroid after staining with Calcein AM (C); and the spheroid after treatment with DOX (100 µM, 30 min at 37°C) (D). Scale-bar is 100 µm. Optical microscopy (A, B) and fluorescence confocal microscopy (С, D).

Multicellular tumor spheroids were generated in polyelectrolyte alginate-oligochitosan microcapsules as described earlier.^20^ MCF-7 cells were cultivated within the microcapsules for 1–3 weeks until they completely filled the inner microcapsule room. A mean microcapsule diameter was 400±50 µm and a membrane thickness was 50±10 µm (Figure 4A-B). The 100% viability of the cells in the spheroids was revealed after homogeneous Calcein AM staining (Figure 3C). As seen in Figure 3C, there were no PI-stained dead cells detected. These observations were in a good agreement with previously reported results which predicted a necrotic core only in the spheroids, which were larger than 500 µm.^22^ As seen in Figure 3D, low molecular weight native DOX easily penetrated through the alginate-oligochitosan membrane. Therefore, we suggest that the microencapsulated spheroids could be used for testing DOX derivatives as well as other low molecular weight compounds.

Microencapsulated tumor spheroids from MCF-7 cells were used to assess cytotoxicity of the obtained DOX derivatives. Cell viability in spheroids was evaluated by MTT assay after 48 and 72 h of incubation of spheroids treated with the DOX derivatives at 37°C (Table 2). As seen in Tables 1 and 2, the tumor spheroids were more resistant to the drug treatment compared to the monolayer culture. The lower IC_50_ value was obtained for native DOX, while the Palm-N_2_H-DOX conjugate was found to be the most effective among all DOX derivatives. For both tumor spheroids and monolayer culture, the cytotoxic effects were found to increase with the enhancement of the incubation time. We should also note that various drugs can be more effective in different models of drug resistance. For example, DOX conjugates with AMG and TPP were more cytotoxic for MCF-7/ADR cells in monolayer culture, than in spheroids. Contrary, the conjugates with 5FU and Palm were more efficient in spheroid model, than in monolayer culture of MCF-7/ADR cells.

**Table 2:** The IC_50_ values of the DOX derivatives in the microencapsulated tumor spheroids from MCF-7 cells.

**Samples**	**IC**_50_ **, µM**	
	**48 h**	**72 h**
**DOX**	26.2	8.1
**Palm-N**_2_ **H-DOX**	37.7	23.0
**N-Palm-DOX**	86.2	43.0
**DOX-5FU**	93.1	74.8
**DOX-AMG**	171.1	97.2
**DOX-TPP**	183.2	101.1

### Cytotoxicity study of HSA complexes with the DOX derivatives

Since the Palm-N_2_H-DOX conjugate was the most effective among other synthesized derivatives, but suffered from lack of solubility, two non-covalent DOX-Palm derivative complexes with HSA, namely HSA-Palm-N_2_H-DOX and HSA-N-Palm-DOX, were prepared. Indeed, these complexes were found to demonstrate excellent solubility in aqueous media without addition of any other solvents like DMSO. Cytotoxicity effects of HSA-Palm-N_2_H-DOX and HSA-N-Palm-DOX were evaluated by MTT assay in monolayer culture for both MCF-7 and MCF-7/ADR cells. In case of MCF-7 cells, the IC_50_ value of 51.4 µM was found for HSA-N-Palm-DOX complex after 72 h of incubation, while about 80% of alive MCF-7/ADR cells were observed even at HSA-N-Palm-DOX concentration of 100 µM. In case of HSA-Palm-N_2_H-DOX conjugate, the IC_50_ values of 35.9 µM, 7.2 µM, and 6.2 µM after 24 h, 48 h, and 72 h of incubation, respectively, were determined. The corresponding IC_50_ values for MCF-7/ADR cells were 39.9 µM, 30.1 µM, and 16.8 µM. It should be noted that these were the minimum values found for the DOX derivatives in this study.

## Discussion

The resistance of tumor cells to chemotherapeutics is a complex phenomenon and one of the major challenges in cancer therapies. At cellular level, multiple drug resistance can arise from the previous exposure to the cytotoxic agents by a number of different mechanisms, including altered efflux the low-molecular drugs from the cells, decreased drug influx, blocked apoptosis, and many others.^23^ At tissue level cellular resistance to drugs is a result of cell-to-cell and cell-to-matrix interactions as well as diffusion limitations caused by 3D architecture of the tumor.^24^ In our study, the synthesized DOX derivatives were proposed to overcome MDR on both cellular and tissue levels using two approaches based on 2D and 3D cell cultures, respectively. First approach was related to the use of MCF-7/ADR cells which is DOX-resistant subline of MCF-7 cells with a high resistance index of 40.8. Second approach was based on the microencapsulated multicellular tumor spheroids, which can mimic some cell-to-cell and cell-to-matrix interactions in small-size solid tumors. Recently, we have demonstrated that cells in microencapsulated spheroids were more resistant than those in monolayer culture against both free drugs^25^ and nano-sized drug delivery systems, namely docetaxel-loaded nanoemulsions or methotrexate prodrug liposomal formulations.^26^ In the current study, RI of the cells in the microencapsulated tumor spheroids against native DOX was found to be approx. 20-fold higher compared to that in the monolayer cell culture. Therefore, we decided to use this 3D *in vitro* model, in order to estimate cytotoxicity effects of the obtained DOX derivatives.

**Figure 5:**
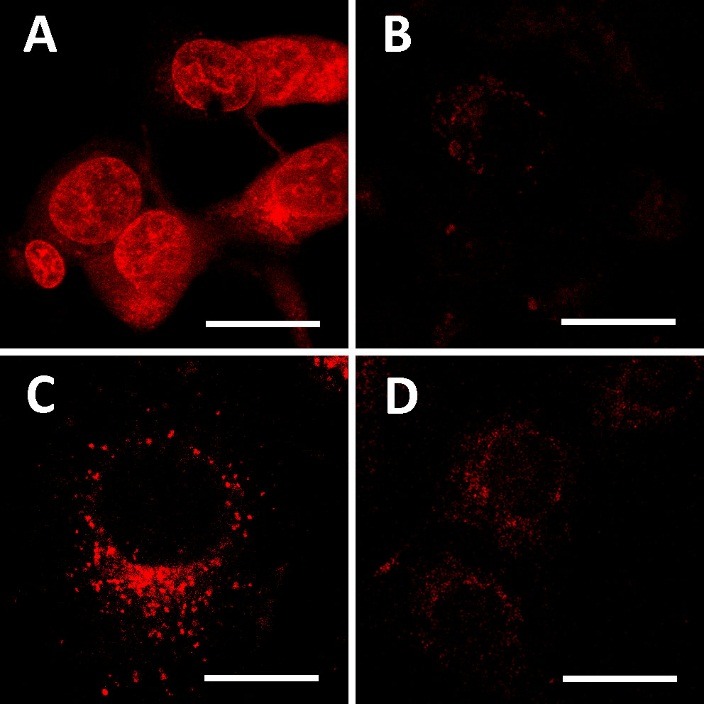
Confocal images of MCF-7 cells after treatment with DOX (A), DOX-TPP (B), DOX-AMG (C), and MitoTracker Orange (D) for 30 min at 37°C. Scale bar is 20 µm.

Our first drug candidate DOX-TPP was aimed at better cell membrane penetrating and mitochondrial targeting due to triphenylphosphonium cation effect.^27^ Mitochondria are the promising target for antitumor treatment because of the lack of efficient DNA repair mechanisms and a key role of mitochondria in the ATP production.^28^ The absence of P-gp transporters which are responsible for MDR in mitochondrial membranes was also demonstrated earlier.^29^ The efficacy of the mitochondrial targeting strategy using TPP against MDR has been reported earlier.^30^ Moreover, mitochondrial delivery of DOX modified with triphenylphosphonium cation was found to lead to drug resistance overcoming in DOX-resistant MDA-MB-435 cells.^31^ In our study, we did not observe neither enhanced cytotoxicity of DOX-TPP nor higher intracellular accumulation for both MCF-7 and MCF-7/ADR cells. However, conjugation with TPP indeed resulted in conjugate exclusion from cell nuclei and possible localization within mitochondria (Figure 5). Therefore, we can suggest that a difference between our results and the data reported by Han at al. ^31^ could be related to alterations in drug resistance developed in MCF-7 and MDA-MB-435 cell lines.

Conjugation of DOX with AMG did not result in increased DOX-AMG uptake compared to native DOX by MCF-7 cells, while it was 2.1-fold higher than that of native DOX in MCF-7/ADR cells. This could be explained by the more efficient delivery of the DOX-AMG conjugate across cell membrane and a bypass of efflux pumps overexpressed in MCF-7/ADR cells. Moreover, the DOX-AMG was successfully delivered to the nucleus in MCF-7/ADR cells. Thus, as we expected, RI determined for this derivative was more than 10-fold lower compared to that in case of native DOX, suggesting a possible potential of this conjugation strategy in MDR-overcoming. However, AMG coupling did not lead to the IC_50_ decrease, and this derivative was the least effective in 3D model based on tumor spheroids.

As well known, 5-fluorouracil is the first rationally designed antimetabolite inhibiting the thymidylate synthase enzyme essential for DNA synthesis and repair.^32^ Since a combination of separately administrated 5FU and DOX has been already successfully used in clinics,^33,34^ the conjugation of these drugs through hydrazone linkage was of great interest. However, cellular uptake of the DOX-5FU conjugate was approx. 5-fold lower compared to that of free DOX for both MCF-7 and MCF-7/ADR cells. This finding could be explained by involvement of specific membrane transporters, in particular uracil transporters involved in 5FU uptake. Unlike in case of MCF-7 cells, in MCF-7/ADR cells DOX-5FU was able to reach nucleus. This could be attributed to a possible difference in DOX-5FU trafficking in cells. In monolayer culture, the IC_50_ values for DOX-5FU conjugate were 17-fold higher than that for native DOX, while in tumor spheroids appropriate IC_50_ values were demonstrated 4- and 10-fold increase after 48 h and 72 h, respectively. We can suggest that this enhancement of DOX-5FU derivative cytotoxicity could appear in 3D model due to more acidic pH in the spheroid center compared to common monolayer culture. Moreover, 5FU uptake was found to increase under acidic conditions, while DOX uptake was decreased.^19,35^ When we compared the IC_50_ values for MCF-7 cells, DOX-5FU conjugate was shown to have a shade higher efficacy compared to that of free 5FU (7.1 and 8.4 µM after 72 h incubation, respectively), but nevertheless it was still approx. 20-fold less cytotoxic than native DOX.

The Palm-N_2_H-DOX and N-Palm-DOX conjugates were aimed to prolong a DOX circulation in the bloodstream due to the complex formation with serum albumin. Since an antitumor effect of palmitic acid was also demonstrated earlier,^36^ we suggested that it could contribute to the cytotoxicity reveled in our study. The cytotoxicity of DOX derivatives with palmitic acid for 2D monolayer culture was reported by Liang et al earlier.^37^ In the current study, we confirmed these cytotoxicity effects using tumor spheroids as 3D in vitro model. Additionally, we studied penetration and accumulation of these DOX derivatives in spheroids. Actually, Palm-N_2_H-DOX which demonstrated the lowest IC_50_ values, the highest uptake level, and accumulation in nucleus of DOX-resistant cells, was found to be the most effective drug candidate for MDR overcoming among all other conjugates studied. It should be noted that another palmitic acid conjugate, namely N-Palm-DOX, demonstrated lower cytotoxicity and reduced penetration. This decrease in cytotoxicity could be related to the decrease of DOX binding to DNA due to the alteration of amino sugar moiety, as reported earlier.^38^ Thus, the anticancer effect of Palm-N_2_H-DOX could be explained not only by the ligand type, but also by the conjugate structure. To release DOX from the conjugate by the cleavage of the amide-bound, lysosomal enzymes are needed, while the hydrazone-bound DOX could be released by pH-sensitive hydrolysis at acidic intracellular conditions.^39^

It has been demonstrated that higher doses of the DOX derivatives were needed in spheroids to achieve the effect similar to that obtained in monolayer 2D model. In both models Palm-N_2_H-DOX was the most effective DOX derivative in terms of MDR overcoming. As well known, *in vivo* transport of long chain fatty acids, including palmitic acid, is mediated by albumin, which is the most abundant plasma protein.^40^ Indeed, in our study, the increase of Palm-N_2_H-DOX and N-Palm-DOX cellular accumulation in the presence of serum proteins was demonstrated *in vitro*. Thereby, the complexes of DOX-palmitic acid conjugates with HSA could be proposed as promising drug candidates for further investigation. Both HSA-Palm-N_2_H-DOX and HSA-N-Palm-DOX complexes were water soluble (up to concentration of 200 µM at least), unlike the Palm-N_2_H-DOX and N-Palm-DOX conjugates which precipitated in DMEM. The HSA-Palm-N_2_H-DOX complex was found to be the most effective drug candidate against MCF-7/ADR cells. This could be explained by albumin macromolecular structure (Mw 66 kDa), which provided the protection against MDR proteins. Finally, we suggested that the HSA-based complexes could provide an advantage over the non-modified Palm-N_2_H-DOX conjugates *in vivo* due to the improvement of the pharmacokinetic profile and higher accumulation level in solid tumors.

## Conclusion

In this study, five DOX derivatives, including 2 novel compounds were synthesized. Cytotoxicity of these DOX derivatives was evaluated using 2D monolayer culture and 3D *in vitro* model based on microencapsulated tumor spheroids. MTS were generated by long-term cultivation of tumor cells in polyelectrolyte alginate-oligochitosan microcapsules. It was demonstrated, that in the case of tumor spheroids the higher doses of all DOX derivatives were needed to achieve the effect similar to that in monolayer culture. Palm-N_2_H-DOX conjugate was found to be the most promising against DOX-resistant MCF-7/ADR cells both in monolayer culture and in tumor spheroids. The formation of non-covalent complex of Palm-N_2_H-DOX conjugate with HSA allowed to improve drug solubility, and as a result to increase its anti-proliferative activity against both MCF-7 and MCF-7/ADR cells.

## Acknowledgments

The study was partly supported by Russian Science Foundation (project 14-13-00731). The authors also would like to thank Drs. E. Svirchevskaya and E. Kovalenko (Institute of Bioorganic Chemistry RAS, Moscow, Russia) for their help and valuable advices.

## Ethical Issues

Not applicable.

## Conflict of Interest

The authors declare no conflict of interests.

## Abbreviations

MDR – multiple drug resistance

2D - two-dimensional

3D - three-dimensional

MTS - multicellular tumor spheroids

RGD - arginine-glycine-aspartic acid

EPR - enhanced permeability and retention

DOX – doxorubicin

HSA - human serum albumin

Palm – palmitic acid

TPP - triphenylphosphonium bromide

5FU - fluorouracil

AMG – aminoguanidine
